# Correction

**Published:** 1882-02

**Authors:** 


CORRECTION.—In the last number of the INDEPENDENT PRACTITIONER several serious errors occurred in the rates appended to some of the journals and magazines in our clubbing list; These errors have now been corrected and we again call the attention of our subscribers to the very great advantages offered to those subscribing through the INDEPENDENT PRACTITIONER COMPANY, 1099 Broadway, New York.

